# Feature Reviews on Food Microbiology

**DOI:** 10.3390/foods15071175

**Published:** 2026-03-31

**Authors:** Efstathios Giaouris, Foteini Parlapani

**Affiliations:** 1 Department of Food Science and Nutrition, School of the Environment, University of the Aegean, 81400 Myrina, Lemnos, Greece; 2 Department of Ichthyology and Aquatic Environment, School of Agricultural Sciences, University of Thessaly, 38446 Volos, Greece; fwparlap@uth.gr

The connection between food microbiology, safety, and human health has never been more important to food science, industry, and society. This Special Issue (SI) features 16 peer-reviewed articles that collectively show how microorganisms—ranging from pathogens to beneficial cultures—shape food systems and influence consumer health. The articles cover a wide array of topics, from advanced molecular diagnostics using next-generation sequencing (NGS) and omics technologies to understand microbial communities in food, to innovative preservation methods and the rediscovery of traditional fermented foods, as well as the mechanistic insights into microbial roles in health.

Among the review articles in this collection, Tsouggou and colleagues synthesize recent advances in shotgun metagenomics for cheese microbiology, demonstrating how comprehensive DNA sequencing reveals not just “who is there” but what they are doing—their metabolic capabilities, resistance genes, and contributions to flavor, quality, and safety [Contribution 1]. This approach goes beyond traditional culture-dependent methods by enabling simultaneous taxonomic profiling and functional gene analysis, providing unprecedented insights into microbial ecosystem dynamics during cheese production and ripening [1,2]. Indeed, recent applications of shotgun metagenomics to fermented foods have uncovered substrate-specific microbial communities with distinct carbohydrate-degrading and health-related gene content, underscoring the technology’s utility for both quality control and functional characterization [3]. Interestingly, this paradigm extends to fermented food matrices broadly, where understanding the ecology of microbial communities unlocks both quality consistency and functional benefits [4].

Another important theme throughout this collection is the strategic use of microorganisms to enhance health while decreasing dependence on synthetic inputs. Martín-Miguélez and colleagues compile extensive evidence on lactic acid bacteria (LAB) isolated from traditional dry-cured fermented foods, such as dry-cured fermented sausages and aged cheeses, documenting their probiotic mechanisms—ranging from adhesion and colonization to bacteriocin production and immunomodulation [Contribution 2]. This work builds on growing awareness that traditional dry-cured fermented foods contain LAB strains with proven health-promoting activities [5,6]. Notably, recent studies highlight that LAB from fermented foods improve digestive health, among other systemic benefits, by enhancing insulin sensitivity, managing type 2 diabetes, increasing short-chain fatty acid (SCFA) levels, and influencing gut microbiota composition through the production of organic acids and other compounds that can create an environment inhibiting pathogens [7,8].

*Helicobacter pylori* is one of the most common human pathogens, infecting up to 50% of the world’s population [9]. Schuppler’s investigation of its potential transmission through food highlights the subtle challenges in confirming foodborne pathways for even the most common pathogens [Contribution 3]. Therefore, although the presence of live *H. pylori* bacteria and/or bacterial DNA in food suggests that foodborne transmission is biologically possible, definitive proof that consuming contaminated food causes infection with this pathogen is still lacking, with person-to-person transmission remaining the most clearly confirmed route [10,11]. In fact, research indicates that *H. pylori* can survive in the extracellular environment for long periods, but the minimum infectious dose in humans remains unknown [12,13].

On the other hand, the work on GABA-enriched yogurt by Ushidee-Radzi illustrates the use of precision fermentation in developing functional foods, addressing the growing consumer interest in natural anxiolytic and cardiovascular substances [Contribution 4]. Therefore, this research demonstrates efficient conversion of glutamate to GABA through LAB fermentation, with the addition of simple carbohydrates boosting production while preserving probiotic viability even after simulated gastric digestion, highlighting GABA’s role in creating a less acidic, more hospitable environment for probiotic survival [14,15,16].

García-García and colleagues explore the ‘Criaderas and Solera’ system in Sherry wines, examining biological aging, flor yeast dynamics, industrial applications, and emerging challenges in this unique fermentation process [Contribution 5]. Indeed, the Criaderas–Solera system ensures consistent Sherry quality because flor yeasts switch to oxidative metabolism. This change allows them to break down non-fermentable substances such as ethanol, glycerol, and organic acids while producing important volatile compounds that shape Sherry’s sensory characteristics [17]. This metabolic shift is associated with the formation of a thick surface biofilm (velum), driven partly by FLO11-related adhesion and increased cell-surface hydrophobicity, which promotes yeast clumping and buoyancy at the wine–air boundary [18]. The formed biofilm not only supports yeast survival in ethanol- and acetaldehyde-rich, nutrient-limited environments but also accelerates aging and provides oxidative protection during biological maturation [19].

As antimicrobial resistance (AMR) increases, natural alternatives from plants and other sources gain more attention. Lemoni and colleagues offer a critical review of edible plant extracts used as food preservatives, highlighting the importance of green extraction methods and standardized antimicrobial testing [Contribution 6]. The review carefully assesses environmentally friendly extraction techniques aimed at maximizing the yield of bioactive compounds responsible for strong antimicrobial effects and explores biochemical mechanisms, including disruption of bacterial cell walls and membranes, inhibition of metabolic enzymes, interference with nucleic acid synthesis, induction of oxidative stress, and suppression of quorum sensing and biofilm development. In fact, recent studies show that green extraction methods, such as ultrasound-assisted, microwave-assisted, pressurized liquid, and supercritical fluid extraction, selectively enhance the release of different classes of bioactive molecules based on their physical and chemical properties, yielding high-yield, food-grade extracts with proven antimicrobial activity [20]. Notably, these eco-friendly techniques reduce solvent use, energy consumption, and thermal degradation, while improving compound purity and extract quality. The produced extracts, often rich in phenolics, flavonoids, essential oils (EOs), or other antimicrobial compounds, are increasingly used in meat, seafood, dairy, and fresh food products, aligning with modern demands for clean-label, functional, and sustainable food production [21,22].

Contemporary food microbiology is increasingly linked to human health through the gut microbiota. Nazzaro and colleagues summarize emerging research on how polyphenol-derived metabolites, produced and transformed by the gut microbiota, mediate anti-biofilm effects, immune responses, and neuroprotection against age-related neurological decline [Contribution 7]. This microbe-driven transformation of dietary components represents a paradigm shift: foods contain not only direct bioactives but also precursors whose full potential is unlocked through microbial metabolism [23]. Accordingly, polyphenols are hydrolyzed by intestinal enzymes or gut microbiota, resulting in metabolites that differ from those in food, which then enter the bloodstream, tissues, and brain, where they influence biological processes by modulating the gut microbiota–brain axis [24,25]. Notably, polyphenol metabolites produced by gut microbiota are well absorbed in the intestine and remain longer in the plasma after conjugation [26]. Additionally, certain polyphenols can inhibit or promote the growth of specific bacteria, thereby altering gut microbial composition to favor beneficial bacteria with higher glycan-degrading enzyme activity [27].

Barros and colleagues provide crucial mechanistic insights into the virulence of enterotoxigenic *Escherichia coli* (ETEC), supporting the development of improved risk mitigation strategies [Contribution 8]. The research explains how ETEC produces heat-labile and heat-stable enterotoxins that attach to specific receptors on epithelial cells. Indeed, colonization factors play a key role in ETEC binding to the intestinal epithelium before toxin delivery, leading to water and electrolyte secretion into the intestinal lumen and causing diarrhea [28]. More specifically, the heat-labile toxin is transported via a Sec-dependent protein secretion system through the bacterial double membrane, while other virulence factors, such as the EatA serine protease, help ETEC access intestinal epithelial cells by degrading the mucus layer [29]. This knowledge offers a deeper understanding of the pathogenesis of this important foodborne pathogen, aiding the development of preventive strategies to reduce ETEC infections and lessen the need for clinical intervention in exposed consumers. Additionally, this article highlights a significant shift in food safety science: moving from simply detecting presence or absence to understanding the mechanisms behind pathogenic processes.

The valorization of food waste through fermentation, as demonstrated by Marcelli and colleagues, shows how food microbiology can effectively address sustainability and nutrition by transforming vegetable processing residues into nutrient-rich, bioactive ingredients [Contribution 9]. This circular approach aligns with global demands for lowering environmental impact and promotes the transition to bioeconomy models. Indeed, fermentation enhances digestibility, reduces undesirable anti-nutritional compounds, and introduces probiotics that benefit human health. It also increases the bioavailability of bioactive compounds, such as antioxidants, vitamins, and prebiotics, enabling the development of innovative functional foods [30]. Notably, according to the Food and Agriculture Organization (FAO), losses in the fruit and vegetable sector can reach up to 60%, making fermentation a promising strategy for converting these by-products into high-value functional ingredients that support more sustainable and circular food systems [31].

Historically, bread was leavened using a natural starter created from the spontaneous fermentation of a mixture of flour and water, driven by naturally occurring bacteria and yeasts. This starter, known as sourdough because of its low pH, is among the oldest and most resilient examples of natural fermentation in food manufacturing and remains a subject of active research [32]. Coronas and colleagues’ discussion of sourdough microbiome preservation and the role of microbial biological resource centers (mBRCs) emphasizes how microbial diversity, now recognized as a form of intangible cultural heritage and genomic resource, requires active protection and management [Contribution 10]. In fact, traditional type I sourdoughs are being rediscovered for their unique flavor, texture, potential health benefits, and extended shelf life for baked goods. However, continuous backslopping preservation may, over time, lead to taxonomic and functional changes in microbial communities, resulting in significant deviations in the characteristics of baked products [Contribution 10]. The identification of appropriate preservation methods, implementation of well-defined access and benefit-sharing protocols, and development of microbiome-specific datasets within mBRCs are crucial for preserving the microbiota of fermented foods, serving as key elements for innovation and safeguarding traditional foods and culinary heritage [33].

Botulism is a rare yet potentially fatal disease that causes paralysis and is caused by neurotoxin produced by *Clostridium botulinum*, an anaerobic, spore-forming bacterium [34]. Amar’s thorough epidemiological review of UK botulism cases highlights that, despite strict food safety measures, rare but serious microbial threats still require ongoing surveillance and quick diagnostic responses [Contribution 11]. These results underscore the importance of integrating real-time diagnostics, understanding the pathogen’s biology, and maintaining flexible food safety regulations. From 2006 to 2009, the UK reported no cases of foodborne botulism. However, between 2010 and 2024, there were 13 cases across seven separate incidents and two outbreaks, with no fatalities. Most cases involved imported, homemade, and artisanal foods, with some linked to commercial products. Challenges in diagnosis and public health include delayed clinical identification, late sample collection, small specimen volumes, and the common unavailability of suspected food sources, all of which complicate epidemiological investigations. Although the UK’s incidence of foodborne botulism remains very low, about 0.001 cases per 100,000 people annually, foodborne botulism continues to pose a public health threat that requires rapid treatment and coordinated regulatory response. These UK epidemiological data, reported challenges, and improvement proposals are certainly helpful to food professionals, diagnostic laboratories, and regulatory authorities in many other countries as well.

Nascimento and Barros’ synthesis of sustainable innovations in food microbiology highlights how traditional practices, combined with modern technology, can promote food security while respecting cultural identity and environmental limits [Contribution 12]. The review critically examines recent advances focused on three main themes: microbial fermentation for sustainable food production, emphasizing the use of agro-industrial waste and the development of functional foods; microbial biocontrol as an eco-friendly alternative to chemical preservatives, including bacteriocins, protective cultures, bacteriophages, and CRISPR-Cas technologies; and functional foods designed to enhance health outcomes through probiotics, prebiotics, synbiotics, and postbiotics, which can impact gut microbiota and support metabolic, immune, and cognitive health [Contribution 12]. This comprehensive review shows that food microbiology remains essential for developing healthier, more sustainable, and science-based food systems, directly supporting the achievement of various United Nations Sustainable Development Goals (SDGs) [35]. In fact, microbial processes such as fermentation, biocontrol, waste valorization, and microbiome-driven agricultural innovations enhance food safety, nutritional value, and resource efficiency [36].

Bacteriophages (phages) are viruses that infect and spread among bacteria. They possess a complex structure featuring a protein coat that encloses their genetic material, which may be DNA or RNA [37]. Trzaskowska’s risk profile of bacteriophages—the first of its kind conducted according to FAO/WHO and EFSA methodologies—demonstrates scientific rigor in evaluating novel antimicrobials, addresses concerns about horizontal gene transfer and resistance development, and affirms the safety potential of phages when appropriately applied [Contribution 13]. This work provides essential evidence of risk assessment that bacteriophages have a dual nature, serving as potential biocontrol agents against pathogenic bacteria and as possible contributors to bacterial adaptation through transduction of virulence and antimicrobial resistance genes [38,39]. This underscores the importance of comprehensive genomic characterization before their use in the food chain [40]. It remains clear that although there are theoretical risks of horizontal gene transfer, properly selected and characterized lytic phages that lack lysogenic genes pose minimal safety concerns. In fact, regulatory frameworks are increasingly recognizing the potential of phage preparations when thoroughly screened for toxin genes, antimicrobial resistance determinants, and temperate features [41].

The linear polymer polyphosphate (polyP) exists in all three domains of life and serves various physiological functions, including phosphorus storage, chaperone activity, and stress tolerance [42,43]. The review by Corrales and colleagues discusses polyphosphate synthesis in LAB and emphasizes that this microbial metabolite impacts intestinal health and food preservation [Contribution 14]. In fact, recent evidence confirms that polyphosphate can positively influence human intestinal health through its homeostatic properties at the intestinal epithelium, generating increased interest in polyphosphate production by probiotic LAB, while polyphosphate is also widely used in the food industry to enhance food quality, preservation, and nutritional value [44,45].

Edible flowers have traditionally been used as food, flavorings, and garnishes and are now becoming more popular in modern cuisine due to changing consumer preferences for new flavors and healthier diets [46]. They improve dish presentation and provide bioactive compounds, including polyphenols, flavonoids, antioxidants, and vitamins, that offer recognized health benefits. Carboni and colleagues provide a comprehensive review of edible flowers, emphasizing their microbiological, nutritional, and potential health benefits, as well as their growing role in functional food development [Contribution 15]. However, microbiological safety remains critical, as edible flowers can harbor microorganisms, including pathogenic bacteria such as *Bacillus* spp., *Enterobacter* spp., *Salmonella* spp., and *Staphylococcus aureus*, as well as mycotoxin-producing fungi such as *Aspergillus*, *Penicillium*, *Alternaria*, and *Fusarium* [47,48]. Therefore, practicing good agricultural methods, maintaining hygienic handling, and proper storage are essential for reducing contamination and ensuring safety. Notably, to our knowledge, there is still no official worldwide list of safe edible flowers, highlighting the lack of international regulatory oversight [49].

Fokas’s investigation into combining EOs with bacteriophages offers promising pathways for enhanced biocontrol, though the authors rightly emphasize challenges related to sensory acceptance and regulatory approval [Contribution 16]. The rise in antimicrobial resistance among foodborne pathogens has increased the search for alternative biocontrol strategies, including EOs that weaken bacterial defenses and make bacteria more susceptible to phage infection and bacteriophages that target specific bacteria and deliver lytic effects against resistant strains. This combined approach improves food safety and supports One Health principles [Contribution 16]. For example, recent experimental data show that oregano EO and lytic bacteriophages work together to combat various foodborne bacteria, lowering antimicrobial levels needed, reducing negative sensory impacts, and providing broad-spectrum activity against both Gram-positive and Gram-negative bacteria [50,51,52]. However, validation in food matrices and confirmation of EO-phage compatibility under intended-use conditions are required before any practical implementation [53].

This SI compilation highlights several key needs. Firstly, standardizing methods for metagenomics, antimicrobial testing, and functional food assessment is crucial for ensuring reproducibility and regulatory uniformity. Secondly, strengthening interdisciplinary collaboration is vital, as food safety increasingly depends on chemists, toxicologists, microbiologists, informaticians, and clinicians working together. Thirdly, regulatory frameworks must evolve to support innovation, as technologies such as bacteriophages, precision fermentation, and new microbial strains show great potential, yet regulatory pathways remain inconsistent across regions. Additionally, this collection demonstrates that microorganisms are no longer viewed just as hazards but as essential partners in developing safe, nutritious, and sustainable food systems. Advances such as genomic pathogen surveillance, probiotic culture application, and natural antimicrobials are transforming food safety, quality, and health promotion. The articles offer both fundamental knowledge and practical insights for food scientists, regulators, producers, and public health professionals dedicated to creating resilient, health-promoting food systems. Future research should focus on integrating omics technologies with real-world validation, developing standardized protocols for international trade and safety, and exploring how microbial communities in food and the human body contribute to sustainable nutrition and public health.

## Data Availability

No new data were created.
